# Identification and Characterization of Secondary Wall-Associated NAC Genes and Their Involvement in Hormonal Responses in Tobacco (*Nicotiana tabacum*)

**DOI:** 10.3389/fpls.2021.712254

**Published:** 2021-09-14

**Authors:** Na Xu, Lin Meng, Lin Song, Xiaoxu Li, Shasha Du, Fengqin Hu, Yuanda Lv, Wenjing Song

**Affiliations:** ^1^Key Laboratory of Tobacco Biology and Processing, Ministry of Agriculture, Tobacco Research Institute, Chinese Academy of Agricultural Sciences, Qingdao, China; ^2^Shandong Provincial Key Laboratory of Biochemical Engineering, College of Marine Science and Biological Engineering, Qingdao University of Science and Technology, Qingdao, China; ^3^Excellence and Innovation Center, Jiangsu Academy of Agricultural Sciences, Nanjing, China

**Keywords:** NAC transcription factors, secondary cell wall, hormonal and stress responses, transcriptional activation, subcellular localization, *Nicotiana tabacum*

## Abstract

Secondary wall-associated NAC *(SWN)* genes are a subgroup of NAC (NAM, ATAF, and CUC) transcription factors (TF) that play a key role in regulating secondary cell wall biosynthesis in plants. However, this gene family has not been systematically characterized, and their potential roles in response to hormones are unknown in *Nicotiana tabacum*. In this study, a total of 40 *SWN* genes, of which 12 from *Nicotiana tomentosiformis*, 13 from *Nicotiana sylvestris*, and 15 from *Nicotiana tabacum*, were successfully identified. The 15 *SWNs* from *Nicotiana tabacum* were further classified into three groups, namely, vascular-related NAC domain genes (*NtVNDs*), NAC secondary wall thickening promoting factor genes (*NtNSTs*), and secondary wall-associated NAC domain genes (*NtSNDs*). The protein characteristic, gene structure, and chromosomal location of 15 *NtSWNs* (also named *Nt1* to *Nt15*) were also analyzed. The *NtVND* and *NtNST* group genes had five conserved subdomains in their N-terminal regions and a motif (LP[Q/x] L[E/x] S[P/A]) in their diverged C- terminal regions. Some hormones, dark and low-temperature related *cis*-acting elements, were significantly enriched in the promoters of *NtSWN* genes. A comprehensive expression profile analysis revealed that *Nt4* and *Nt12* might play a role in vein development. Others might be important for stem development. Quantitative reverse transcription-polymerase chain reaction (qRT-PCR) revealed that in the *NtNST* group, genes such as *Nt7, Nt8*, and *Nt13* were more sensitive than the genes in *NtVND* and *NtSND* groups under abiotic stress conditions. A transactivation assay further suggested that *Nt7, Nt8*, and *Nt13* showed a significant transactivation activity. Overall, *SWN* genes were finally identified and characterized in diploid and tetraploid tobacco, revealing new insights into their evolution, variation, and homology relationships. Transcriptome, *cis*-acting element, qRT-PCR, and transactivation assay analysis indicated the roles in hormonal and stress responses, which provided further resources in molecular mechanism and genetic improvement.

## Introduction

Secondary cell walls are mainly made up of xylem treachery elements and fibers (Zhong and Ye, 2015a), which are present in the plant cells but absent in the animal cells. They can protect plant cells from potential biotic and abiotic stresses (Brown et al., 2005). They are also the main component of plant materials used for fuel, paper-making, and textiles (Zhong et al., 2010). Secondary wall-associated NAC *(SWN)* genes are one of the largest transcription factor (TF) families (Su et al., 2013). Among the NAC family, vascular-related NAC domain proteins (VND1 to VND7) and NAC secondary wall thickening-promoting factor proteins (NST1, NST2, and NST3) play a key role in secondary wall biosynthesis (Zhong and Ye, 2015a). Besides, two *Arabidopsis SWN* domain genes, SND2 and SND3, are also specifically expressed in secondary cell wall development (Hussey et al., 2013; Zhong and Ye, 2014). After a comparative genome analysis across 19 higher plant species, SWN transcriptional factors, including three groups, SND, NST, and VND, were identified as playing a crucial and ruling role in secondary cell wall biosynthesis (Yao et al., 2012).

In *Arabidopsis thaliana, AtSWN* genes have been proved to be key regulators of secondary cell wall biosynthesis (Wang and Dixon, 2012). *AtVND1* to *AtVND5* are specifically expressed in xylem vessels, and they can activate the expression of genes involved in secondary wall biosynthesis (Zhou et al., 2014). *AtVND6* and *AtVND7* can control the formation of metaxylem and protoxylem vessels, and their over-expression will result in the ectopic deposition of the patterned secondary cell walls (Ohashi-Ito et al., 2010). *AtNST1* and *AtNST2* are redundantly responsible for secondary wall thickening in anther endothecium. *AtNST1* and *AtNST3* redundantly regulate the secondary wall thickening in the secondary xylem of hypocotyls (Mitsuda et al., 2007). In addition, *AtNST1, AtNST2*, and *AtSND1* regulate secondary cell wall biosynthesis in fibers of stems (Zhong et al., 2006; Zhong and Ye, 2015b). *AtSND2* and *AtSND3* are shown to promote secondary wall thickening in fibers (Grant et al., 2010). *AtVND1–AtVND7* binds directly to the SNBE/TERE-like motif in the *AtVND7* promoter region (Nakano et al., 2015). *AtVND1–AtVND5* probably triggers xylem tracheary element formation by regulating *AtVND7* expression (Fukuda, 2016). *AtSND3* is the direct target gene of *AtNST1, AtNST1, AtNST2, AtVND6*, and *AtVND7* (Zhong et al., 2008). *AtSND2* is indirectly regulated by *AtSND1*/*AtNST1*, but there is no evidence for regulation by *AtVND6*/*AtVND7* (Hussey et al., 2011). Transactivation assays showed that the expression of *AtSND1* operates under positive feedback control from itself and that the AtSND1 protein could bind directly to a conserved motif in its promoter (Wang et al., 2011). A two-hybrid assay of yeast revealed the ability of *AtVND7* to form homodimers and heterodimers with other *AtVND* proteins (Yamaguchi et al., 2008). These results indicate that secondary wall biosynthesis is a complex and delicate process; *AtSWN* genes from the NAC family are key TFs.

Hormones and many TFs also directly regulate or indirectly influence *SWN* genes. In *Populus* stems, auxin can repress *SND/NST* genes associated with fiber and secondary wall formation but induce vessel-specific *VND* genes; GA (gibberellic acid) induces the expression of *SWN* genes (Johnsson et al., 2019). In *Arabidopsis*, auxin inhibits fiber wall thickening by affecting *LBD* (LATERAL ORGAN BOUNDARIES DOMAIN) genes and repressing the expression of *AtNST1*- *AtNST3* (Lee et al., 2019). LBD15 can directly bind with the upstream sequence of the *AtVND7* gene and positively regulate *AtVND7* expression (Ohashi-Ito et al., 2018). *AtSND1* and *AtNST2* are upregulated in the mutants of *athb15*, which codes a TF that belongs to the HD-ZIP III family and functions as a suppressor of the secondary wall-related regulatory pathway (Du and Wang, 2015). *WRKY12* can bind directly to the promoter of *AtNST2* to repress its expression, resulting in the suppression of secondary wall biosynthetic genes in pith cells (Wang et al., 2010). E2Fc, a member of the E2F family, can activate the expression of *AtVND7* in a dose-dependent manner (Rao and Dixon, 2018). MYB26 is capable of binding to the promoter of *AtNST1* and *AtNST2* for the regulation of secondary wall thickening (Yang et al., 2007). MYC2 and MYC4 from the bHLH family can also directly bind the promotor region of *AtNST1* (Zhang et al., 2018).

In this study, we identified a total of 40 secondary-wall associated NAC transcriptional factors from *Nicotiana tabacum* (K326), *Nicotiana sylvestris*, and *Nicotiana tomentosiformis*. We analyzed 15 *NtSWN* genes from *N. tabacum* for further research. After successfully cloning these *NtSWN* sequences, their gene structures were analyzed, such as motifs, chromosome localization, and expression patterns in different tissues. Additionally, we also analyzed the *cis*-acting elements of their promoter regions and how they respond to hormones and stress. Finally, functional characterization of the motifs, transactivation activity assay, and subcellular localization were analyzed and studied as well.

## Materials and Methods

### Plant Materials and Hormone Treatments

*Nicotiana tabacum* (K326) was used in all the experiments. The seedlings were grown in a culturing room at 25°C under a 16 h:8 h light/dark cycle. To clone the sequences of *NtSWNs* (Supplementary Table 4), a mixture of complementary DNA (cDNA) extracted from the root, stem, and leaf of 4-week-old seedlings were used as an amplification template. Forward/reverse primers (Supplementary Table 3) were designed in the nearest upstream/downstream regions close to the start/stop codon of the coding sequences (CDS). To obtain correct cDNA sequences, the 2 × Taq Plus Master Mix (Dye Plus) (P213-01; Vazyme Biotech Co. Ltd., Nanjing, China) was used to amplify the *NtSWNs*, and sequenced with Sanger technology. To investigate the response of *NtSWN* genes to hormones, 3-week-old seedlings, under normal conditions, were separately given 10 μM GA (GA_3_, G8040; Solarbio, Beijing, China), 200 μM abscisic acid (ABA, A8060; Solarbio, Beijing, China), 2 mM methyl salicylate (MeSA, SB8540; Solarbio, Beijing, China) in ultrapure water, 100 μM methyl jasmonate (MeJA, M8640; Solarbio, Beijing, China) in.01% (V/V) ethanol, 0.5 μM α-naphthaleneacetic acid (NAA, N8010; Solarbio, Beijing, China), 0.5 μM 2,4-epi-brassionlade (epiBL, B8780; Solarbio, Beijing, China), dark, and 4°C treatments. The seedlings were collected at 0, 1, 3, 6 h after treatment initiation, and non-treated seedlings were used as a control. The samples were collected and immediately frozen in liquid nitrogen and stored at −80°C for RNA extraction. Three biological replicates were employed per sample.

### Transcriptome Sequencing and Expression Analysis

Total RNA from the root, stem, and leaf of 4-week-old seedlings was extracted with the RNAprep Pure Plant Kit (432; TIANGEN, Sichuan, China) and submitted into ribonucleic acid sequencing using the Illumina HiSeq × Ten system with the paired-end method by Annoroad Gene Technology (Beijing) Co. Ltd. For transcriptome analysis, reference genome and annotation files with a gene model were downloaded from the SOL genomics network (SGN, http://www.sgn.cornell.edu) database directly. Raw reads were trimmed for low quality and length by Fastp v0.20.0 (Chen et al., 2018). Clean reads were aligned to the reference genome using Hisat v2.0.5 (Pertea et al., 2016) with the splice-aware method. The gene expression levels from RNA-seq were quantified by feature counts (Liao et al., 2014) and normalized by fragments per kb per million reads (FPKM) values. Differential expression analysis of two conditions/groups (two biological replicates per condition) was performed using the DESeq2 R package (1.16.1) (Love et al., 2014). The *p*-values were adjusted using Benjamini and Hochberg's approach for controlling the false discovery rate. Genes with an adjusted *p* < 0.05 found by DESeq2 were assigned as differentially expressed. The raw data were deposited in the NCBI SRA database (Accession: PRJNA605912).

### Identification and Phylogenetic Analysis of the NAC Genes in *Nicotiana* spp

The sequences of *N. tabacum* (K326), *N. sylvestris*, and *N. tomentosiformis* were downloaded from the SGN database (*Solanaceae* Genomics Network, https://solgenomics.net/organism/Nicotiana_tabacum/genome) (Edwards et al., 2017). Previously reported AtSWN protein sequences (AtSWNs) (Zhou et al., 2014) were retrieved from The *Arabidopsis* Information Resource (TAIR, http://www.arabidopsis.org) database, and then used as seed sequences to blast against the SGN database with the parameter *E*-value of 10^–5^. After manually removing the redundant sequences, candidate SWN protein sequences with a conserved domain (PF02365) were analyzed with the NCBICDD online program (https://www.ncbi.nlm.nih.gov/Structure/bwrpsb/bwrpsb.cgi). Multiple sequence alignments of the SWN protein sequences at the amino acid level were performed using the ClustalX program. A neighbor-joining (NJ) phylogenetic tree was then generated based on the alignment result using MEGA 7.0 with the following parameters: Poisson model, pairwise deletion, and bootstrap values (1,000 replicates) (Kumar et al., 2016). Chromosomal locations were determined based on the chromosomal information derived from the SGN database. The positions of the *NtSWN*s were physically mapped to each chromosome according to their coordinates on the tobacco genome. Finally, *SWN* genes in *N. tabacum* (*NtSWNs*) were identified and named based on their physical locations across the chromosomes and scaffolds.

### Gene Structure, Conserved Motif Analysis, and *cis*-Acting Regulatory Element Analysis

The number of amino acids, molecular weight (MW), and isoelectric point (pI) for each SWN protein were calculated with the DNAStar software (Version 7.0) (Burland, 2000). The subcellular localizations were predicted with ProtComp 9.0 (http://linux1.softberry.com). Exon-intron structures were analyzed by comparing the CDSs and genomic sequences obtained from the SNG database. Conserved motifs were identified with Multiple Em for Motiv Elicitation (MEME) 5.0.5 (Bailey et al., 2015). Parameters were set as follows: distribution of motif occurrences, zero or one per sequence; maximum number of motifs, 25; optimum motif width, ≥10 and ≤24; sites per motif, ≥2 and ≤14. All the results, together with the NJ tree, were visualized with TBtools V0.665 (Chen et al., 2020). The upstream region sequences (3,000 bp) for each gene were also extracted from genome sequences and used for *cis*-acting element analysis using the Plant CARE database (http://bioinformatics.psb.ugent.be/webtools/plantcare/html/) (Lescot et al., 2002).

### Quantitative Reverse Transcription-Polymerase Chain Reaction (qRT-PCR) Analysis

First-strand cDNA synthesis was performed using 1 μg total RNA with the HiScript®IIIRT SuperMix for qPCR [+ genomic DNA (gDNA) wiper] (R323-01; Vazyme Biotech Co. Ltd., Nanjing, China). The qRT-PCR was performed on a QuantStudio™ 3 Real-Time PCR System machine in a 20 μl reaction with ChamQ^TM^ Universal SYBR® qPCR Master Mix (Q711-02/03, Vazyme Biotech Co. Ltd., Nanjing, China). The qRT-PCR assay results were obtained from three independent replicates. Expression was calculated using the 2^−ΔΔCT^ method (Livak and Schmittgen, 2001) and normalized by comparison with the *NtActin* gene (Accession:XM_016618658.1).

### Transactivation Activity Assay

The CDS of *Nt7, Nt8, Nt10*, and *Nt13* were amplified using cDNA from K326 leaves with gene-specific primers (Supplementary Table 6). The PCR products were combined into the *pBD-GAL4* vector *via* EcoRI/SalI sites with specific primers (639636; Clontech, CA, United States) (Xiao et al., 2018), and with the *pBD-GAL4* vector as the negative control. The yeast strain AH109 (YC1010; Weidi, Shanghai, China), containing the *HIS3, ADE2*, and *MEL1* reporter genes, was used to test transcriptional activation activity. These constructs, together with *pGADT7*, were transfected into AH109 following the instructions of the yeast strain manufacturer. The transformed yeast cells were incubated on a synthetic dropout (SD) medium lacking leucine and tryptophan (DDO, SD/-Leu-Trp) plates at 30°C for 4 days. Then, the positive colonies were transferred to the SD medium lacking leucine, tryptophan, histidine, and adenine (QDO, SD/-Leu-Trp-His-Ade) supplemented with X-α-Gal plates at 30°C for 4 days.

### Subcellular Localization

The full length of *Nt7* and the NAC domain of *Nt8* and *Nt13*, excluding the terminator codon, was amplified and ligated into the subcellular localization vector pYG57, which contains the CaMV35S promoter and a C-terminal green fluorescent protein (GFP) domain. Positive clones were confirmed by DNA sequencing, and the recombinant constructs and control were transformed into *Agrobacterium tumefaciens* strain GV3101. Forty-eight hours after *Agrobacterium*-mediated transient expression in the 4-week-old *N. benthamiana* seedlings, fluorescence signals were captured with a confocal microscope (TCS-SP8; Leica, Wetzlar, Germany). DNA dye 4,6-diamidino-2-phenylindole (DAPI) staining was performed to show the position of the nucleus.

## Results

### Genome-Wide Identification of SWN Family Genes in Tobacco

Twelve AtSWN proteins were employed to search for potential SWNs against the tobacco database by blast and hmmsearch programs. Redundant sequences were then removed through manual reconstruction. Based on conserved domains, the protein sequences shared the same clades, and the AtVND, AtNST, and AtSND groups were used for phylogenetic analysis. A total of 40 NtSWN proteins were successfully identified, including 12 from *N. tomentosiformis* (Nto), 13 from *N. Sylvestris* (Nsy), and 15 from *N. tabacum* (Nt), which were further renamed as Nto1 to Nto12, Nsy1 to Nsy13, and Nt1 to Nt15 (Supplementary Table 1). The full-length amino acid sequences of NtSWNs varied from 279 to 399, and the average amino acid length was 339. The MWs of NtSWN proteins ranged from 31.6 to 45.5 kDa, with the average being 38.9 kDa. The pI values ranged from 5.11 to 8.89, with an average of 6.6. The predicted sub-cellular localization revealed that all the NtSWN proteins were localized to the nucleus (Supplementary Table 2). Furthermore, the *NtSWN* genes were aligned to the reference genome by the blast program and only 7 of the 15 *NtSWN* genes were successfully mapped on six chromosomes. *Nt4* and *Nt5* genes were localized on chromosome 17, and five other *NtSWNs* were mapped on Chr4, Chr6, Chr12, Chr22, and 23 (**Figure 3C**).

### Phylogenetic Analysis

**Figure 1:**
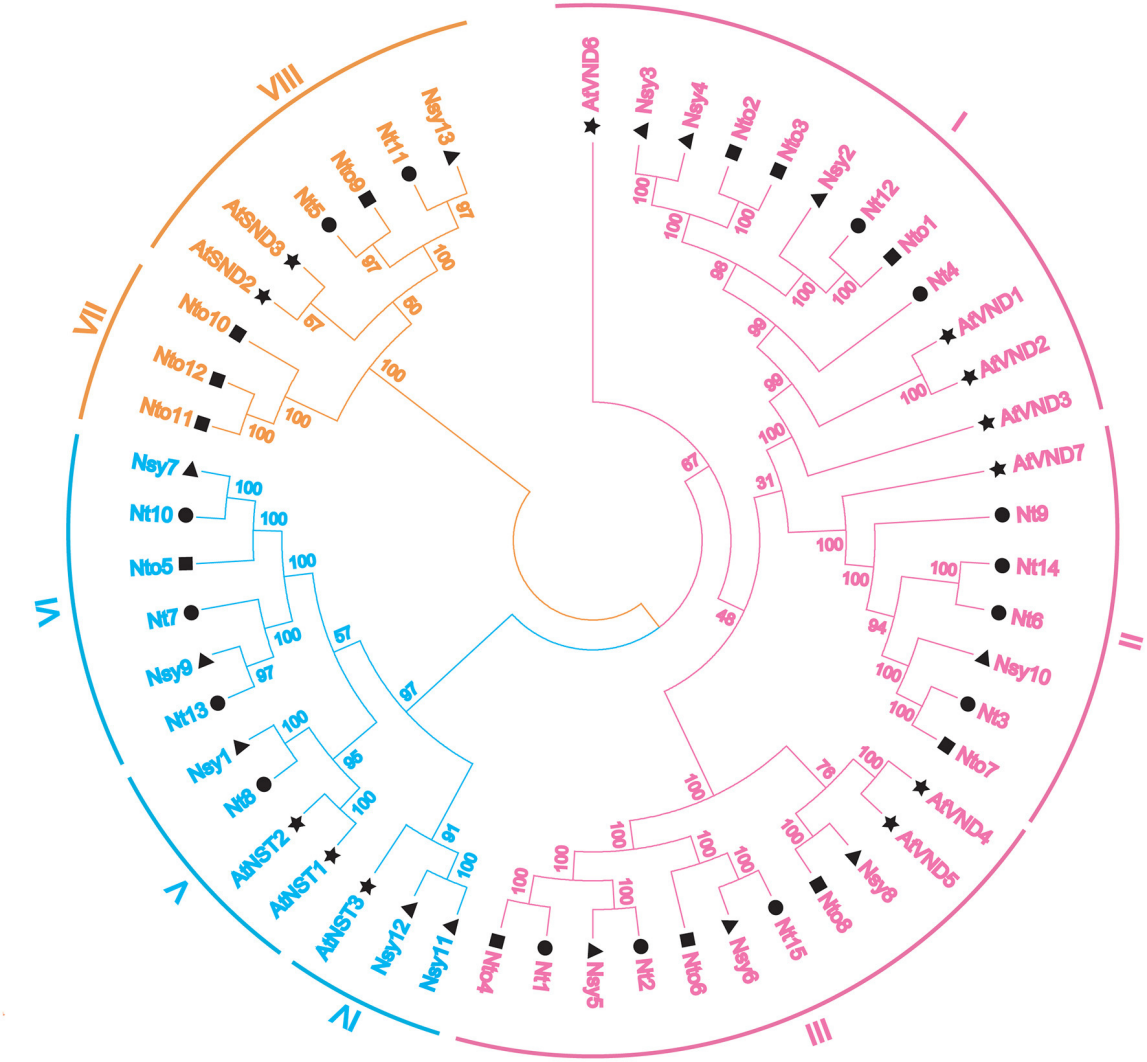
Phylogenetic tree of secondary wall-associated NAC genes (SWNs) with *Arabidopsis thaliana, Nicotiana tomentosiformis, Nicotiana sylvestris*, and *Nicotiana tabacum*. Fifty-two SWN proteins were classed into three groups, which are highlighted in specific colors: vascular-related NAC domain genes (VNDs) in pink, NAC secondary wall thickening promoting factor genes (NSTs) in blue, and secondary wall-associated NAC domain genes (SNDs) in orange. Black graphic symbols represent different species: stars indicate *A. thaliana*, triangles indicate *N. Sylvestris*, squares indicate *N. tomentosiformis*, and circles indicate *N. tabacum*. Peripheral arcs indicated the subgroups of each group, and Roman numerals on the arc represent the subgroups of each group.

To investigate the evolutionary relationships among the NtSWN proteins, 52 SWN proteins from *A. thaliana, N. tomentosiformis, N. sylvestris*, and *N. tabacum* were aligned by the ClustalX program with multiple alignment method. A neighbor-joint tree was then generated by the MEGA7 program (Figure 1). Based on the phylogenetic tree, the SWN proteins could be clustered into three groups, corresponding to VND, NST, and SND. Every group was highlighted with different colors. The NtSWN proteins from the three tobacco species could be further classed into eight subfamilies (I–VIII) (Figure 1). Among them, the VND and NST groups were categorized separately into three subgroups, while the SND group was divided into two subgroups.

### Exon–Intron Organization of *SWN* Genes

**Figure 2:**
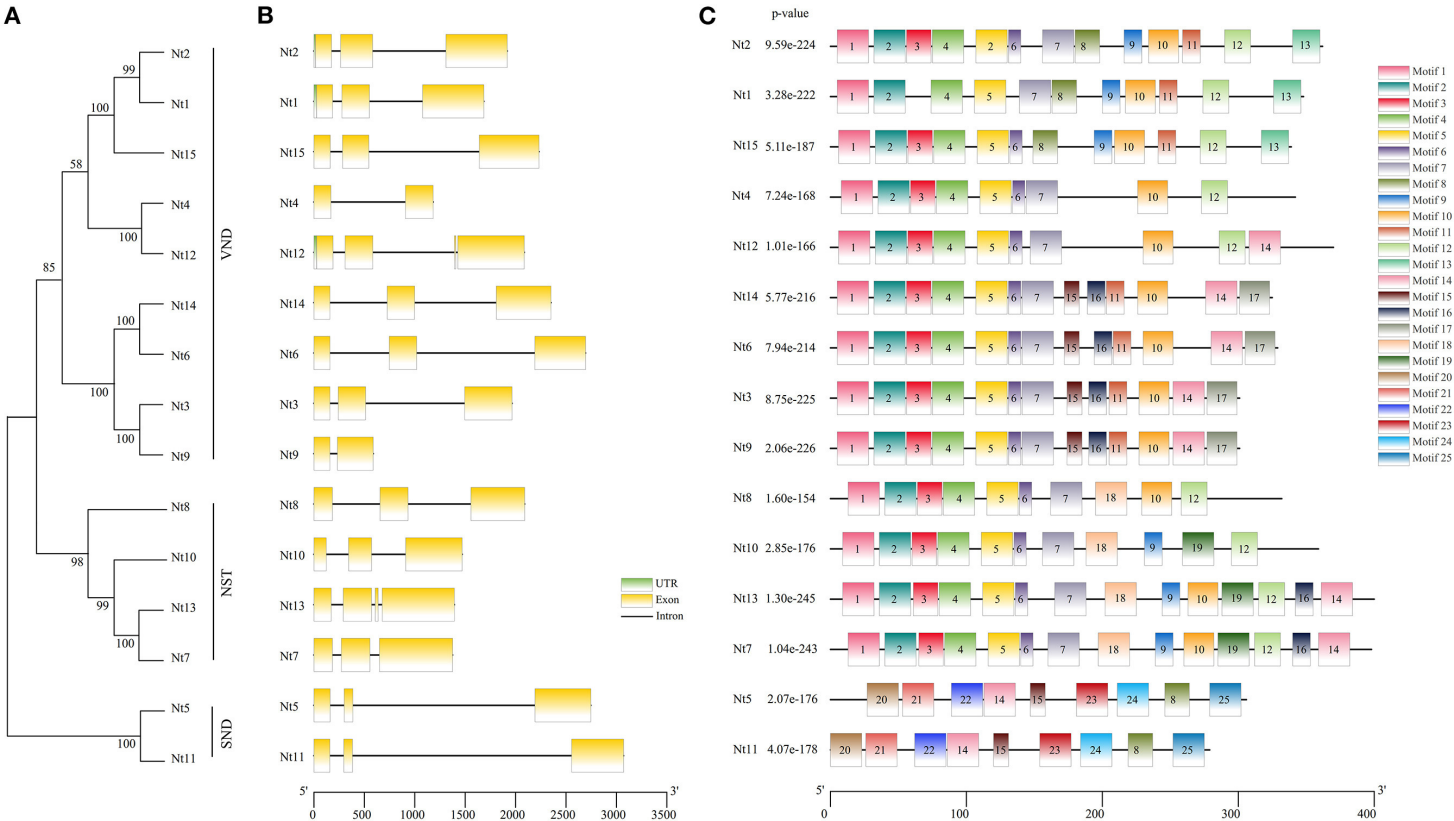
Neighbor-joining (NJ) phylogenetic relationships, gene structure, and motif analysis of NtSWNs. **(A)** In the phylogenetic tree, the NtVND, NtNST, and NtSND groups were marked in a straight line on the right. **(B)** In gene structures, untranslated region (UTRs) were indicated by green boxes, exons were represented by yellow boxes, and introns were shown in black lines. **(C)** Motif compositions were detected with Multiple Em for Motiv Elicitation (MEME); in motif analysis, the motifs were represented by different colored boxes, and each gene contained 9 to 14 motifs.

To gain further insight into the phylogenetic relationship among the 15 *SWN* proteins from *N. tabacum*, a NJ tree was generated individually (Figure 2A). The phylogenetic tree was also classed into three groups, homologous to AtVND, AtNST, and AtSND groups. Patterns of exon–intron architecture variation of the *NtSWN* genes are analyzed, as shown in Figure 2B. Most of *NtSWN* genes had three exons and two introns, but *Nt4* and *Nt9* genes had two exons, and *Nt13* had four exons. The length of the second intron of genes in the NtSND subgroup was ~2 kb, which was longer than what was found in the other genes (Figures 2A,B). The genes in the same subgroup generally shared similar exon/intron structures. These results showed that *NtSND* and *NtNST* genes were more conserved in the exon–intron structure than *NtVND*, and showed the gene structure divergence of different subfamilies during evolution (Figure 2C).

### Conservation and Divergence of C-Terminal Domain Structure in NtSWN Proteins

**Figure 3:**
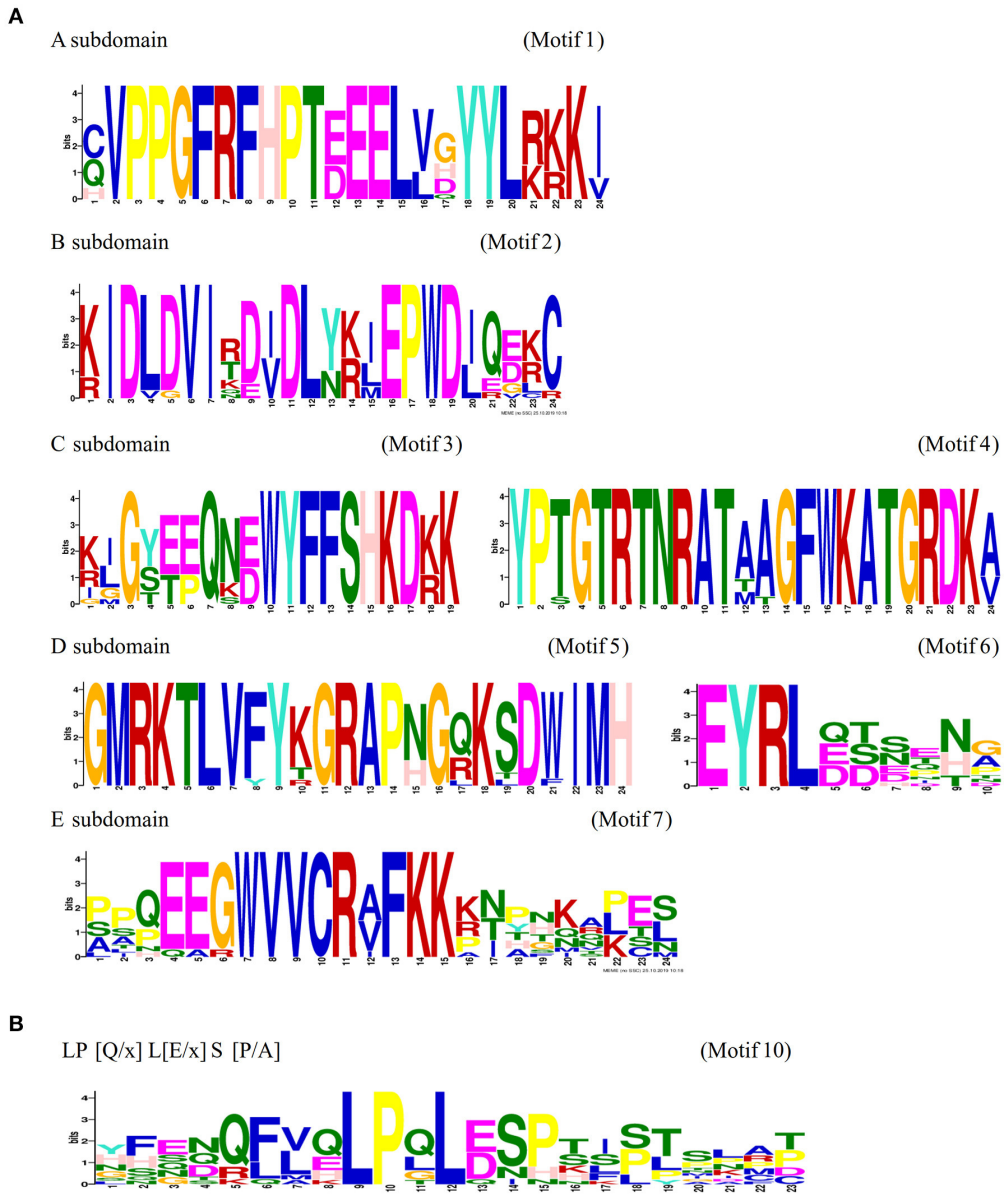
Sequence-specific MEME motifs for NtVNDs and NtNSTs. **(A)** A–E subdomains were marked in the top left corner, and their corresponding motif numbers are shown in Figure 2C were indicated in the top right corner. **(B)** The LP [Q/x] L[E/x] S [P/A] motif, namely motif 10, is also shown.

A typical NAC protein comprises a conserved N-terminal NAC domain (~150 amino acids) and a diversified C-terminal transcription regulatory (TR) region (Puranik et al., 2012). The NAC domain contains five subdomains, namely, A, B, C, D, and E (Satheesh et al., 2014), and the NtVND and NtNST groups have conserved motif patterns, which apparently constitute a conserved NAC domain. In the N-terminal region of NAC, seven motifs were identified in the NAC domain corresponding to subdomains A, B, C, D, and E, and the amino acids in these motifs were conserved (Figure 3A). Among them, Nt1 was without motif 3 in the C subdomain and motif 6 in the D subdomain and Nt15 was without motif 7 in the E subdomain. Compared with the N-terminal, the C-terminal of genes in the NtVND and NtNST groups were highly diverged. The type, quantity, and distribution of the motifs were relatively conserved only in the same subgroup. Motif 18 existed in all NtNSTs and was unique to this group. Except for Nt10, NtVNDs and NtNSTs had motif 10 (Figure 3B). Interestingly, although the C-terminal was highly divergent, motif 10 (LP[Q/x] L[E/x] S[P/A]) was the highly conserved motif across many species (Shen et al., 2009), which indicated that it might be a functional element. The NtSND group was clustered into an independent branch, which is highly divergent in the NAC domain. NtSNDs had nine motifs, such as motifs 14, 8, and 15, which were common with those motifs in the TR region of the NtVND and NtNST groups, while six other motifs were NtSND-specific. These suggested that potential functional differences in NtSNDs existed.

### *cis*-Acting Element Analysis of *NtSWN* Genes

**Figure 4:**
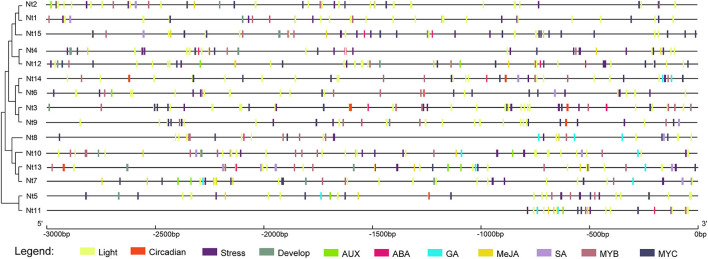
Distribution of main *cis*-acting elements in the 3 kb upstream promoter regions of *NtSWN* genes. The different types of *cis*-acting elements were represented using rectangles with different colors. The scale above was used to measure nucleoside length.

Promoter regions that contain many *cis*-acting elements play important roles in the regulation of gene expression response to stresses (Ding et al., 2016). The 3 kb upstream regions of *NtSWNs* were submitted into the PlantCARE database for the detection of useful information on the regulatory mechanism (Figure 4). All the 15 *NtSWN* genes contained light-responsive, abiotic stress-related, and hormone-responsive elements. Except for *Nt6, Nt8, Nt9, Nt11*, and *Nt14*, the other 10 gene promoters had development-related elements. Furthermore, only *Nt3, Nt9, Nt13*, and *Nt14* had circadian control elements. These results implied that *NtSWNs* were related to plants responding to light, stress, hormone, and development, and that *NtSWNs* could be regulated by TFs. The circadian control elements in *Nt3, Nt9, Nt13*, and *Nt14* gene promoters suggest that they might have a distinct diurnal expression pattern. The most abundant *cis*-acting elements were MYB-related (Myb, MYB, MYB recognition site, Myb-binding site, MYB-like sequence) and MYC-related (Myc, MYC). The highest diverged *cis*-acting elements were light-responsive. Auxin (AuxRR-core, TGA-box, TGA-element), gibberellin (GARE-motif, P-box, TATC-box), ABA (ABRE), MeJA (CGTCA-motif, TGACG-motif), and salicylic acid (TCA-element) responsiveness elements were also found in the promoters of *NtSWN*s, which indicated that auxin, gibberellin, jasmonic acid, and salicylic acid participated in the expression regulation of *NtSWN* genes. The elements involved in ABA and MeJA were the most abundant, followed by ABA and gibberellin elements.

### *NtSWN* Gene Isolation and Expression Pattern in Different Tissues

**Figure 5:**
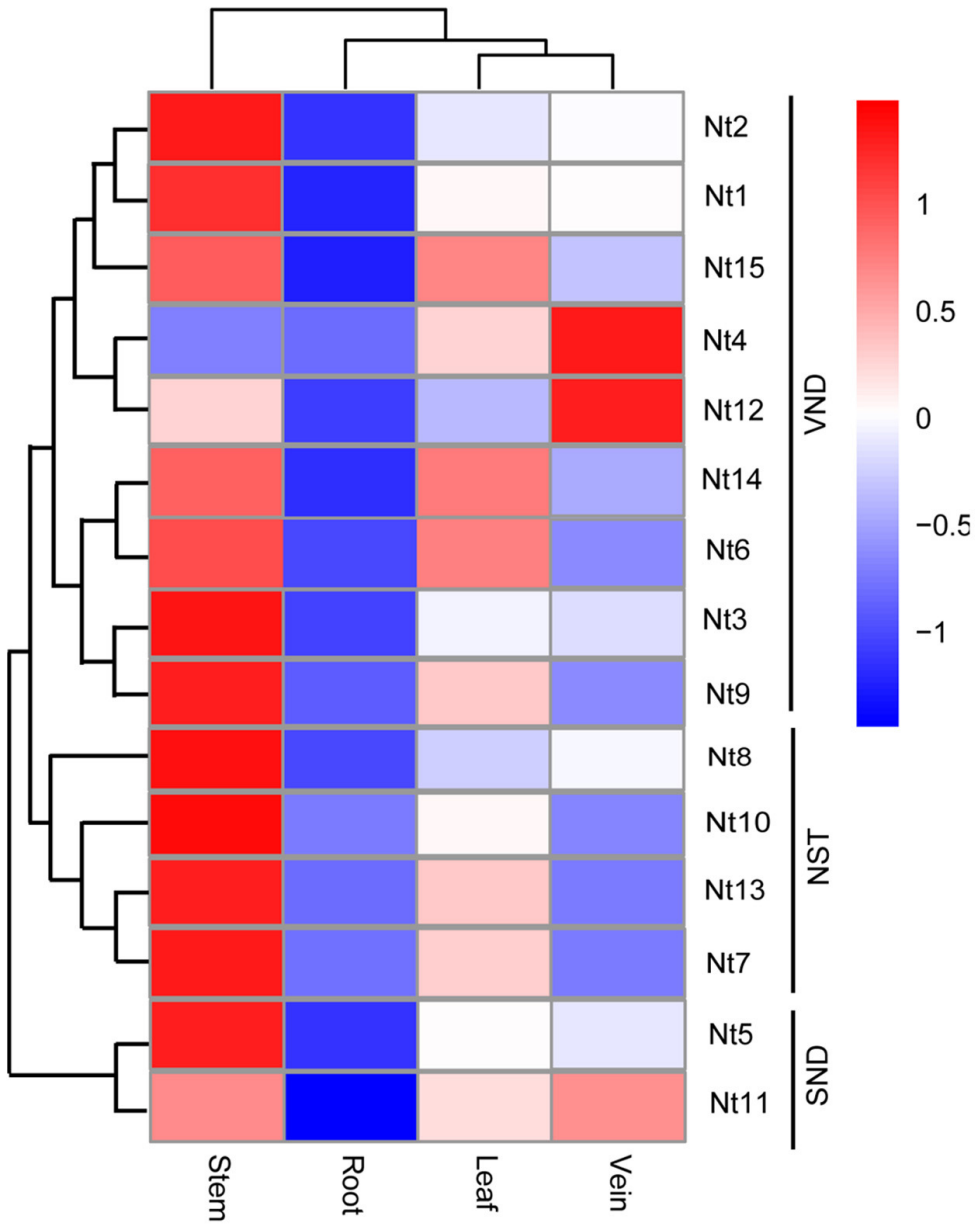
Expression profile of *NtSWN* genes in tissues. Expression patterns of the *NtSWNs* in the root, stem, leaf, and vein of 3-week-old seedlings.

Expression patterns of these genes were then analyzed using the RNA-seq data. Genes in the same subgroup tended to have similar expression patterns (Figure 5). The expression level of *NtSWN* genes was highest in the stem, followed by leaf and vein and in root it was minimum. Interestingly, the expression patterns in the stem and vein of *Nt4* and *Nt12* in the VND group were opposite to the other genes. The gene expression profiles in different tissues provided preliminary clues for their function. These results showed that most *NtSWN* genes are involved in stem development, and that *Nt4* and *Nt12* might participate in vein development. Most of the *NtSWN* genes have a similar expression pattern, which indicated that the secondary cell wall thickening mainly in stem at this development stage, and these transcriptional factors were co-regulated in this process.

### Expression Dynamic of *NtSWN* Gene Response to Hormones and Stresses by qRT-PCR

**Figure 6:**
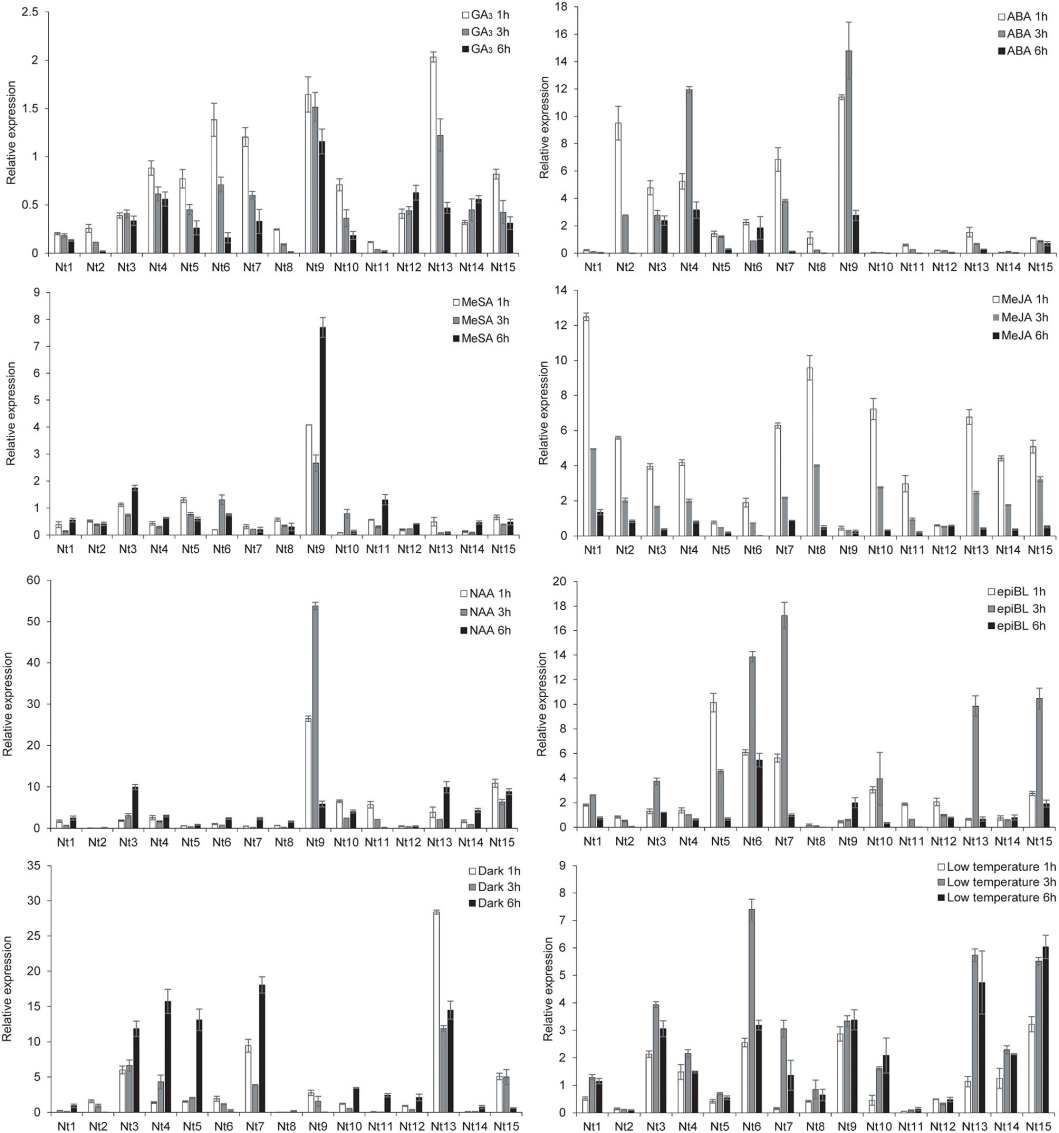
Quantitative reverse transcription-polymerase chain reaction (qRT-PCR) analysis of *NtSWN* expression in response to hormone treatments and abiotic stresses. Tree-week-old seedlings were treated with 10 μM gibberellic acid (GA3), 200 μM abscisic acid (ABA), 2 mM methyl salicylate (MeSA), 100 μM methyl jasmonate (MeJA), 0.5 μM α-naphthaleneacetic acid (NAA), 0.5 μM 2,4-epi-brassionlade (epiBL), dark and 4°C. Error bars indicate SE.

To identify the *NtSWN* genes responsive to hormones and abiotic stresses, the expression patterns of *NtSWNs* in plants subjected to GA, ABA, MeSA, MeJA, NAA, epiBL, dark, and low temperature treatments were investigated by qRT-PCR (Figure 6). The PCR primers used in this study are shown in Supplementary Table 5, and the 0 h seedlings were used as control. In statistics, differential expression patterns of *NtSWNs* were analyzed by *t*-test (Supplementary Table 7). The expression level of most of the *NtSWNs* were upregulated with the ABA, MeJA, NAA, epiBL, dark, and low temperature treatments, but those genes were downregulated with the GA_3_ and MeSA treatments. For the GA_3_ and MeJA treatments, the expression level of most of the *NtSWNs* reached peaks at 1 h and then kept decreasing. In the ABA, epiBL, and low temperature treatments, the expression level of most of the *NtSWNs* reached peaks at 3 h. For the MeSA, NAA, and dark treatments, the expression level of most of the *NtSWNs* reached peaks at 6 h. *Nt9* was remarkably induced by ABA, MeSA, and NAA, when its transcription level increased more than 14-fold at 3 h, 7-fold at 6 h, and 53-fold at 3 h, respectively. In the GA_3_ and dark treatments, the expression level of *Nt13* was upregulated by 2-fold and 28-fold at 1 h, respectively. *Nt1*was upregulated with MeJA by 12-fold at 1 h; *Nt7* upregulated with epiBL by 17-fold at 3 h; *Nt6* was upregulated with low temperature by 7-fold at 3 h. *G*enes in the *NtNST* group were used for further research, because they were more sensitive to hormone and abiotic stresses than those in *NtVND* and *NtSND*.

### Transactivation Activity Assay

**Figure 7:**
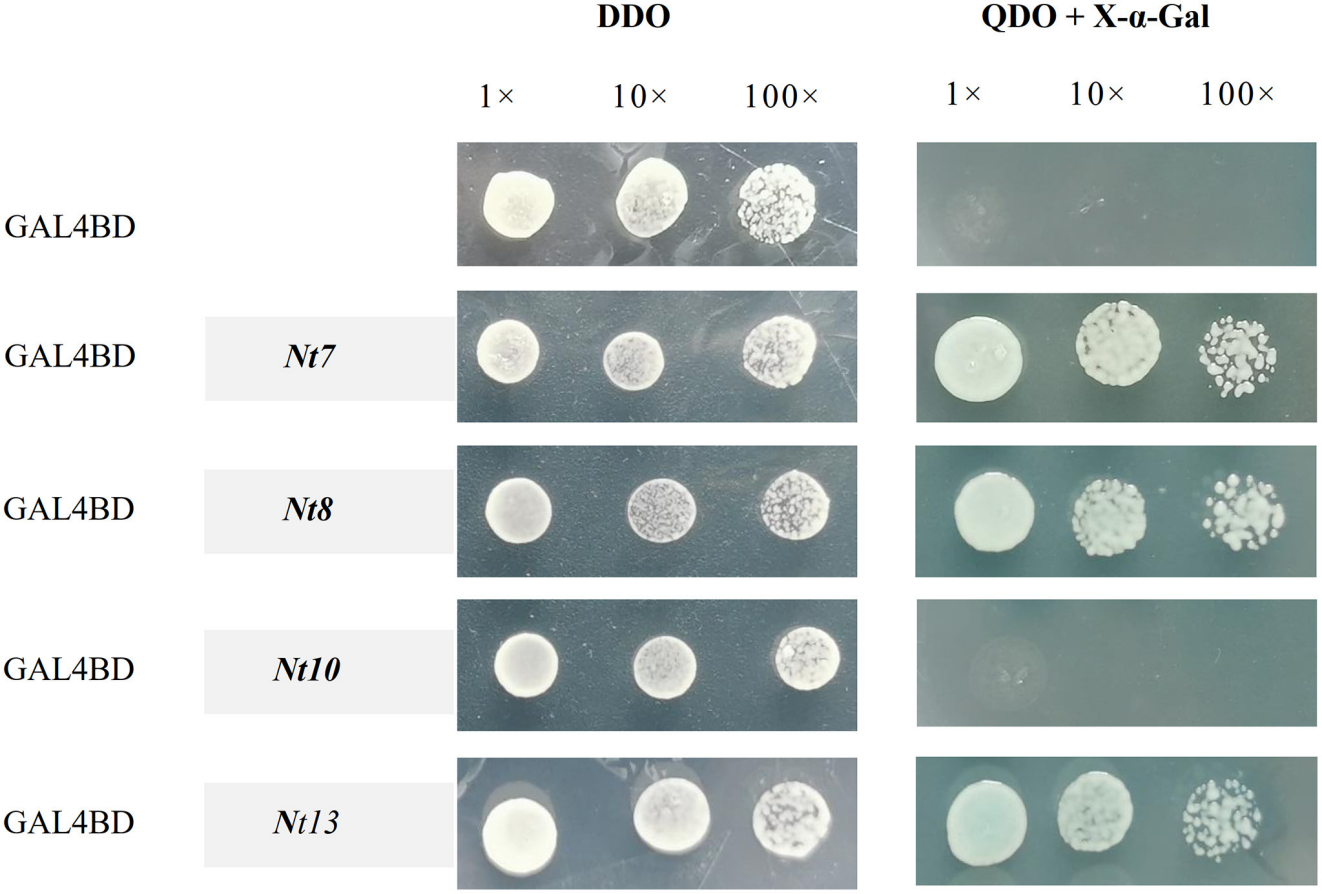
Transactivation analysis of *Nt7, Nt8, Nt10*, and *Nt13*. Genes were combined into the GAL4 (BD) DNA binding domain in pBD-GAL4, with the empty pBD-GAL4 vector as the negative control. The transformed yeast cells were incubated on DDO (SD/-Leu-Trp) and QDO (SD/-Leu-Trp-His-Ade) with X-α-Gal. The yeast was diluted 10 and 100 times.

The full-length CDS of the NtNST group (*Nt7, Nt8, Nt10*, and *Nt13*) were used to investigate the transactivation activity with the reporter genes of AH109, such as *lacZ, HIS3, ADE2*, and *MEL1*. The transgenic yeasts were selected on the SD medium (SD/-Leu-Trp), followed by X-α-Gal activity monitoring on the SD medium (SD/-Leu-Trp-His-Ade). The yeast transformant constructed with *pBD-GAL4-t7, pBD-GAL4-Nt8*, and *pBD-GAL4-Nt13* turned blue in the presence of X-α-Gal and grown normally on the SD medium (SD/-Leu-Trp-His-Ade), whereas the *pBD-GAL4-Nt10* and negative control did not (Figure 7). These results suggested that Nt7, Nt8, and Nt13 could regulate the expression of downstream target genes, and that Nt10 perhaps could not.

### Subcellular Localization

**Figure 8:**
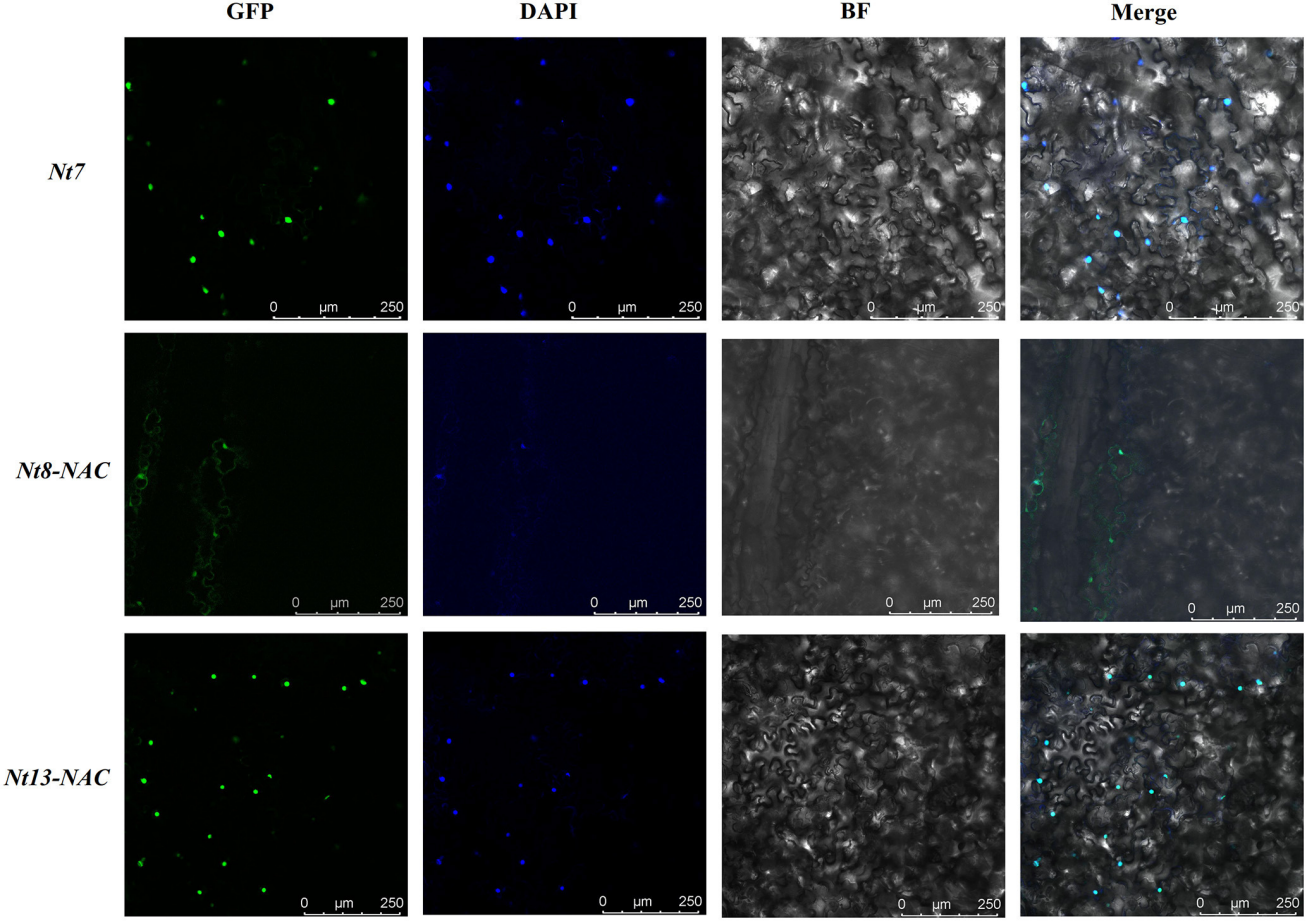
Subcellular localization of *Nt7* and the NAC domain of *Nt8* and *Nt13*. The fusion constructs and green fluorescent protein (GFP) driven by the 35S promoter were transiently expressed in tobacco. DNA dye 4,6-diamidino-2-phenylindole (DAPI) was used as a nucleus marker, and the green fluorescent signals of these genes can be merged with DAPI.

We chose the genes with transactivation activity to further determine their subcellular localization (Figure 8). The open reading frame (ORF) of *Nt7, Nt8*, and *Nt13*, excluding the stop codon, was cloned to be in a frame with a GFP reporter gene, which was under the control of the CaMV-35S promoter. Fusion protein, including *Nt7*-GFP, *Nt8*-GFP, and *Nt13*-GFP with full-length CDS, were constructed. But no GFP signal were observed in *Nt8*-GFP and *Nt13*-GFP. NAC-domain of *Nt8* and *Nt13* were further cloned and inserted into pYG57 vector for subcellular localization. The GFP signals from full-length CDS of *Nt7* and the NAC domains of *Nt8* and *Nt13* were all localized in the cell nucleus and merged with DAPI. These results suggested that fusion proteins were specifically localized in the nucleus.

## Discussion

The major constituent tissue of wood is the xylem, which includes tracheary elements, fibers, and rays (Zhou et al., 2011). Compared with *Arabidopsis*, tobacco has typical xylem (Chaffey et al., 2002) and is easier to do gene transformation. Therefore, tobacco is an ideal model plant in xylem development research (Edwards et al., 2017). Secondary wall formation is an important process in xylem differentiation (Zhong and Ye, 2015a). SWNs are master TFs in the process of secondary wall deposition (Ohashi-Ito and Fukuda, 2010). To date, SWNs have been identified and characterized in many plants; however, few studies on SWNs in tobacco have been reported. As a result, it is of great importance to isolate SWNs from the tobacco genome and explore their characteristics.

*Nicotiana tabacum* is an allotetraploid, and it is generated *via* natural crossing between *N. sylvestris* and *N. tomentosiformis*. In this study, we successfully isolated 15 NtSWNs, and the *N. tabacum* SWNs were fewer probably because of low-sequence conservation or incomplete genome information of *N. tabacum* (Figure 1) (Li et al., 2018). These 15 NtSWNs, together with AtSWNs, divided into VND, NST, SND, suggesting that the ancestors of SWNs differentiated before the divergence between tobacco and *Arabidopsis* (Ding et al., 2016).

The exon-intron organization in the same subgroup was similar, indicating a close evolutionary relationship (Figure 2B). In general, among the NAC proteins of different plant species, NAC domains are conserved when TR regions are diverse. However, in this study, NtSNDs did not have a conserved NAC domain at their N-terminal, while NtVNDs and NtNSTs had conserved patterns at their C-terminal (Figure 2C). The motifs in the N-terminal of NtSNDs were different from the A to E subdomains (Shen et al., 2009). Moreover,motifs A, D, and E were present in all the NAC genes, whereas motifs B and C were absent in several NAC genes, which were grouped together in the phylogeny (Satheesh et al., 2014).

The analysis of *cis*-acting elements helped us to predict the genes responsive to hormones, environment, and stress. Seven kinds of *cis*-acting elements exist in the promoters of *NtSWNs*, such as light-responsive elements, stress-related elements, hormone-responsive elements, development-related elements, circadian control elements, and MYB and MYC elements (Figure 5). The ubiquitous MYB and MYC elements in the promoters of *NtSWNs* suggested that these TFs might be important upstream regulators. MYB46 and MYB83 are direct targets of SWNs in the regulation of secondary cell wall biosynthesis (Zhong and Ye, 2012), but up to now, only MYB26 was reported as the upstream regulatory factor of SWNs (Yang et al., 2007). The ubiquitous MYB elements suggest that there might exist many other MYBs that can directly bind the promoters of *NtSWNs*. Light-responsive elements are the most variable *cis*-acting elements in the promoters of *NtSWNs*, which indicated that different colors and intensities of light might regulate the expression of *NtSWNs* through different pathways.

Different genes had the same *cis*-acting elements in their promoter-region, which might provide clues for further exploration of different molecular functions of *NtSWNs*. For example, three NAC transcription factors, ANAC019, ANAC055, and ANAC072, were proved to bind MYC-like sequence (CATGTG) in *Arabidopsis* (Tran, 2004). Besides, BES1(bri1-EMS-suppressor 1), a positive regulator in brassinosteroid (BR) signaling pathway, interacts with BIM1 (BES1-interacting MYC-like 1) to synergistically bind to E box (CANNTG) sequences present in many BR-induced promoters in *Arabidopsis* (Yin et al., 2005). It was suggested that both NAC and BES1 might regulate the expression of *NtSWNs* by binding to an MYC element. The widespread G-box, mentioned as a light-responsive element, was also the target of BZR1 (an important gene in the BR signal pathway.) (Oh et al., 2012; Zhang et al., 2014). It was suggested that both light and BR could regulate the expression of *NtSWNs* by binding to G-box. Due to BR also playing an important role in photomorphogenesis (Kang et al., 2001, Paulisic et al., 2017), the light might regulate the expression of *NtSWNs* through the BR signal pathway. Moreover, the same factor might regulate the *NtSWNs* genes through different *cis*-acting elements. For instance, jasmonate (JA) and MYC TFs, which emerged as both activators and repressors of the JA signaling pathway, can bind to G-box (Yang et al., 2018). It was suggested that JA might regulate *NtSWNs* through G-box. Besides G-box, JA could regulate *NtSWNs* through the GARE-motif, P-box, and TATC-box as well. Auxin regulates cambial activity; GA can regulate xylem differentiation and xylem fiber elongation (Elo et al., 2009); BR can promote xylem differentiation (Zhang et al., 2011); JA can stimulate secondary growth (Sehr et al., 2010). Cold, light, and salicylic acid can stimulate lignin biosynthesis; BR can regulate cellulose biosynthesis (Didi et al., 2015; Le Gall et al., 2015). VND2, VND4, VND5, and NST3 are upregulated by ABA, and VND6 is downregulated by auxin and inducted by BR (Didi et al., 2015). According to previous studies, we explored the expression profiles of *NtSWNs* comprehensively, and it is the first time to report the expression profiles of *NtSWNs* with different hormones and stresses (Figure 7). Statistical results show that most of *NtSWNs* could be upregulated by ABA, MeJA, NAA, epiBL, dark, and low temperature, and downregulated by GA_3_ and MeSA. With the ABA, MeJA, NAA, and low temperature treatments, the expression pattern of *Nt12* was different from that of the other genes, indicating that the function of *Nt12* in hormonal and stress responses might be opposite to that of the other genes. In comparison with the other genes, *Nt9* was more sensitive to ABA, NAA, and MeSA, indicating that *Nt9* might play an important role in the response to these hormones. The gene expression profiles in different tissues provided preliminary clues for their function. These results showed that most of the *NtSWN* genes are involved in stem development, and that *Nt4* and *Nt12* might participate in vein development. Most of the *NtSWN* genes have a similar expression pattern, which indicated that the secondary cell wall thickening, mainly in the stem at this development stage and transcriptional factors, were co-regulated in this process.

A transactivation activity assay is always performed to investigate whether a gene has transactivation activity and performs the function as a TF in regulating the expression of downstream target genes. Information on the subcellular localization of proteins can help to clarify their functions and be used to predict the subcellular localization of homologous genes in other species. In this study, *Nt7, Nt8*, and *Nt13* localized in the cell nucleus and had a transactivation activity, which indicated that *Nt7, Nt8*, and *Nt13* are functional genes. This analysis provides preliminary results of the putative functions of NtSWNs for further research on the regulation of secondary cell wall biosynthesis and xylem development.

## Conclusions

In this study, a total of 40 *SWN* genes in tobacco were systematically identified and characterized. Fifteen *NtSWN* genes from the NtVND, NtNST, and NtSND groups were further investigated for their expression patterns, dynamic change, and transactivation with hormones and stresses. Transcriptome and qRT-PCR analysis revealed that the NtNST group genes, such as *Nt7, Nt8*, and *Nt13*, were more sensitive to abiotic stresses. The transactivation assay further suggested that *Nt7, Nt8*, and *Nt13* showed a significant transactivation activity. These results will provide useful insights on secondary cell wall-associated NAC genes for further molecular mechanism and genetic improvement in tobacco.

## Data Availability Statement

The datasets presented in this study can be found in online repositories. The RNA-Seq data were deposited in the NCBI SRA database (Accession: PRJNA605912). The accession numbers of NtNAC genes can be found in the article/Supplementary Material.

## Author Contributions

WS and YL conceived and designed the experiments. NX and LM performed the experiments. LS, XL, SD, and FH participated in data collection and analysis. NX wrote the manuscript. WS and YL revised the manuscript. All the authors have read and approved the final manuscript.

## Funding

This study was financially supported by the Agricultural Science and Technology Innovation Program (ASTIP-TRIC03), the Science Foundation for Young Scholars of Tobacco Research Institute of Chinese Academy of Agricultural Sciences (2018B03), and the National Nature Science Foundation of Shandong Province (ZR2019BC066).

## Conflict of Interest

The authors declare that the research was conducted in the absence of any commercial or financial relationships that could be construed as a potential conflict of interest.

## Publisher's Note

All claims expressed in this article are solely those of the authors and do not necessarily represent those of their affiliated organizations, or those of the publisher, the editors and the reviewers. Any product that may be evaluated in this article, or claim that may be made by its manufacturer, is not guaranteed or endorsed by the publisher.

## Supplementary Material

Supplementary Table 1 Protein sequences of the secondary wall-associated NAC genes *(SWN)* gene family members in *Arabidopsis, Nicotiana sylvestris, Nicotiana tomentosiformis*, and *Nicotiana tabacum* (K326).

Supplementary Table 2 Supplementary Table 2. Characteristics of NtSWNs identified in *Nicotiana tabacum*.

Supplementary Table 3 Specific primers for the amplification of *NtSWNs*.

Supplementary Table 4 Complementary deoxyribonucleic acid (cDNA) sequences of *NtSWN* genes.

Supplementary Table 5 Specific primers for quantitative reverse transcription-polymerase chain reaction (qRT-PCR).

Supplementary Table 6 Specific primers for transactivation activity assay.

Supplementary Table 7 Statistical differential expression analysis of 15 *NtSWNs* between treatments and control.
